# WE READ HIS PAL CARD AT THE FUNERAL: EVALUATING THE IMPLEMENTATION OF A PERSON-CENTERED COMMUNICATION TOOL

**DOI:** 10.1093/geroni/igz038.193

**Published:** 2019-11-08

**Authors:** Katherine M Abbott, Howard Degenholtz

**Affiliations:** 1 Miami University, Oxford, Ohio, United States; 2 University of Pittsburgh, Pittsburgh, Pennsylvania, United States

## Abstract

This symposium describes the development and implementation of an interdisciplinary and novel person-centered care (PCC) communication tool in nursing homes (NH). PCC is a philosophy that recognizes “knowing the person” and honoring individual preferences. The communication tool is based on an assessment of NH resident likes and dislikes via the Preferences for Everyday Living Inventory (PELI). The PELI is an evidenced-based, validated instrument that can be used to enhance the delivery of PCC. In 2016, the Ohio Department of Medicaid (ODM) mandated NHs use the PELI as one of the factors that determine the quality portion of their daily Medicaid reimbursement rate. The Preferences for Activity and Leisure (PAL) Card was developed to communicate important resident preferences across care team members. In 2018, the PAL Card Project was approved by the Ohio Department of Aging as a Quality Improvement Project. The first presentation will describe the implementation of PAL Cards with n=43 NH providers. The second presentation will present data regarding the acceptability, feasibility, and appropriateness of the communication tool as rated by providers. The final presentation explores provider qualitative responses regarding the characteristics of the PAL Card communication tool related to effective implementation. The Discussant, Dr. Howard Degenholtz will discuss the implications of initiatives to address the quality of resident care.

